# Discovery of sunflower-derived umami peptides via machine learning and molecular dynamics simulation of their interactions with the T1R1/T1R3 receptor

**DOI:** 10.3389/fnut.2026.1961387

**Published:** 2026-09-25

**Authors:** Zimo Zhang, Fei Ye, Haifeng Zhao, Xiaozheng Shi

**Affiliations:** 1 School of Life Sciences and Biopharmaceuticals, Shenyang Pharmaceutical University, Shenyang, China; 2 School of Biological and Pharmaceutical Engineering, Jilin Agricultural Science and Technology College, Jilin City, China; 3 Jilin Institute for Drug Control, Changchun, China; 4 Changchun University of Chinese Medicine, Changchun, China

**Keywords:** machine learning, molecular dynamics simulation, sunflower, T1R1/T1R3 receptor, umami peptides

## Abstract

**Introduction:**

Umami recognition relies on T1R1/T1R3 binding with umami peptides, but the interaction mechanisms remain unclear. This study aims to reveal the taste mechanisms of four potential umami peptides at both sensory and molecular levels.

**Methods:**

Electronic tongue evaluations were performed to assess umami and richness intensities. Principal component analysis (PCA) was used to discriminate the peptides. Molecular docking and 200 ns molecular dynamics (MD) simulations were conducted to characterize the binding modes and conformational dynamics of peptide–receptor complexes.

**Results:**

Electronic tongue evaluation showed that ELLADMK exhibited the highest umami (1.05) and richness (1.77) intensities, followed by LEELMDK (umami: 0.97), HELDTK (umami: 0.72), and ADVLEK (umami: 0.49). PCA effectively discriminated these peptides, accounting for 93.8% of the total variance (PC1: 71.8%, PC2: 22.0%). Molecular docking and 200 ns MD simulations revealed that umami peptide binding decreased the conformational stability of the Venus flytrap domain, induced conformational expansion (e.g., in the ELLADMK system, RMSD increased from 2–4 Å to 8–10 Å, and Rg increased from 31.0–31.5 Å to 32.0–32.5 Å), and altered solvent accessibility in key regions. Notably, ELLADMK exhibited the most prominent modulatory effect.

**Discussion:**

These findings clarify the sensory and molecular mechanisms underlying umami peptide recognition. The pronounced modulatory effect of ELLADMK suggests its potential as a highly active umami peptide. Overall, this study provides a theoretical basis for the targeted screening of highly active umami peptides and for precise flavor regulation.

## Graphical abstract

**Figure:**
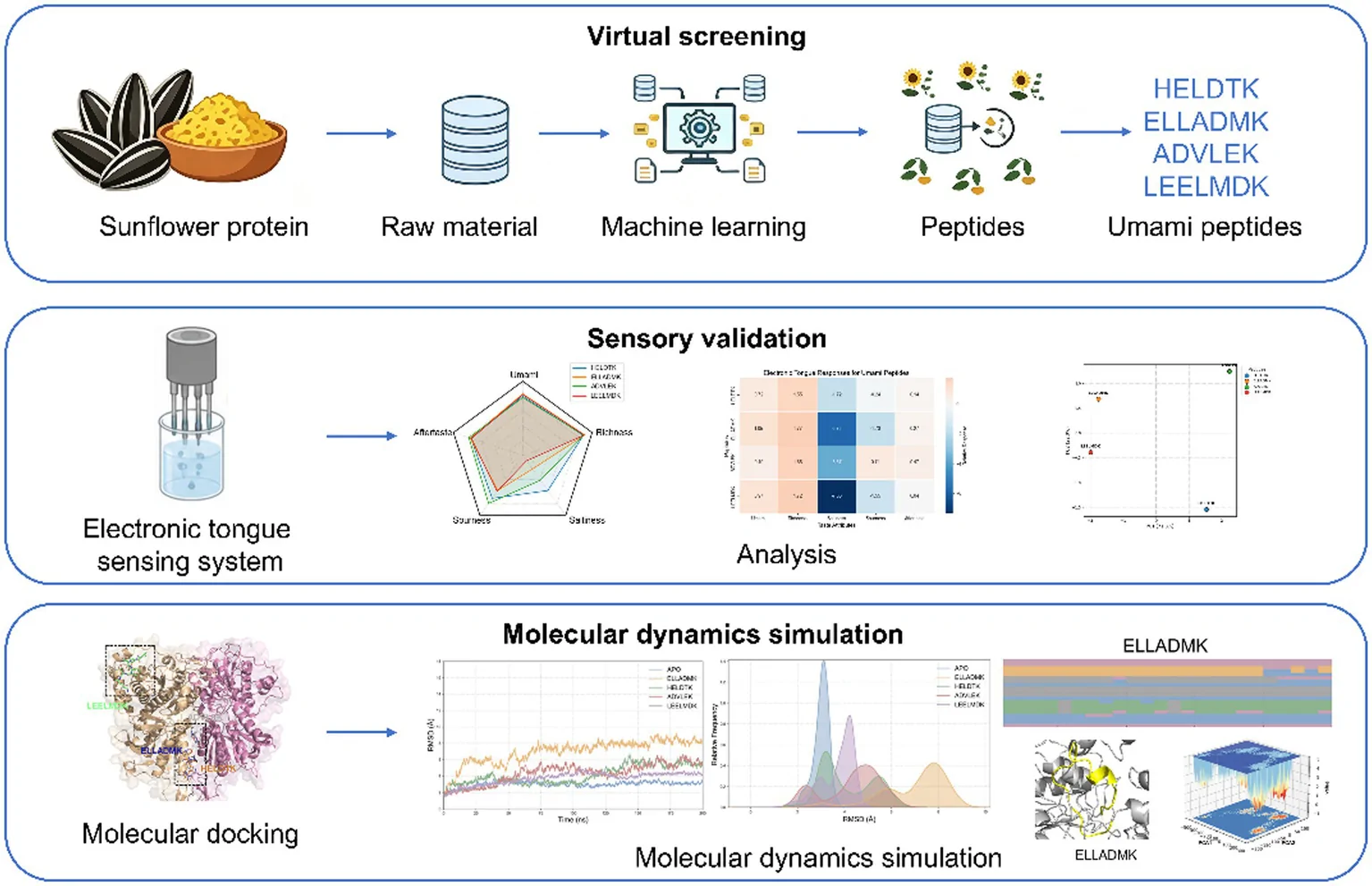

## Highlights

- Machine learning was used to identify high-potential umami peptides from sunflower.
- Sensory profiles of identified peptides were validated by electronic tongue analysis.
- Molecular dynamics revealed key interactions between peptides and T1R1/T1R3 receptor.
- ELLADMK peptide effectively induced structural expansion and flexibility in T1R1/T1R3.

## 1 Introduction

Sunflower (*Helianthus annuus* L.) is one of the most important oilseed crops globally. During the oil extraction process, a large amount of sunflower meal is generated as a primary byproduct (1). Although rich in high-quality proteins and essential amino acids, sunflower meal is conventionally underutilized, often downgraded to animal feed or fertilizer (2). In recent years, the enzymatic hydrolysis of plant proteins to release value-added bioactive (3) and flavor-active peptides (4) has emerged as a promising strategy to valorize byproducts (5). Therefore, exploring sunflower protein as a sustainable precursor for flavor peptides holds significant potential for the food industry.

Umami (6), widely recognized as the fifth basic human taste, plays a crucial role in enhancing food palatability, improving flavor profiles, and potentially assisting in sodium reduction diets (7). The perception of umami is primarily mediated by the specific binding of umami peptides to the T1R1/T1R3 heterodimeric taste receptor, particularly within its Venus flytrap domain (VFD) (8). Unlike traditional umami agents such as monosodium glutamate (MSG) (9), food-derived umami peptides offer complex sensory dimensions, including richness and mouthfulness (10). However, the exact recognition mechanisms and the dynamic conformational changes of the T1R1/T1R3 receptor (11) upon peptide binding remain largely elusive at the molecular level.

Traditionally, the discovery of umami peptides has relied on empirical approaches, such as complex enzymatic hydrolysis followed by extensive separation, purification, and sensory evaluation (10). However, these conventional methodologies exhibit significant drawbacks: they are inherently time-consuming, labor-intensive, and require substantial organic solvents and raw materials. Furthermore, this extraction paradigm is essentially a “blind” trial-and-error process. It not only suffers from low screening efficiency but also risks the loss or degradation of highly active trace components during multi-step purification. In contrast, the targeted screening workflow established in this work offers significant technical advantages to overcome these bottlenecks (12). With the rapid development of bioinformatics and computational chemistry, machine learning (13) and structure-based virtual screening have revolutionized the identification of functional peptides (14). By directly predicting sequence properties and evaluating peptide-receptor binding mechanisms prior to experimental synthesis, this targeted strategy drastically reduces the screening space and accelerates the discovery cycle (15). While artificial intelligence models can efficiently predict potential umami sequences from vast protein databases (16), experimental validation and deep mechanistic exploration are still indispensable to bridge the gap between sequence prediction and biological interaction (17, 18).

To systematically discover and characterize sunflower-derived umami peptides, this study implemented an integrated pipeline combining bioinformatics, machine learning, and experimental validation. Initially, the sunflower (*Helianthus annuus* L.) proteome was retrieved from the NCBI database and subjected to in silico (virtual) hydrolysis to generate a comprehensive peptide library. Subsequently, a machine learning-based screening was employed to identify potential umami sequences from the library. The sensory profiles of the selected candidate peptides (ELLADMK, HELDTK, ADVLEK, and LEELMDK) were then rigorously evaluated using an electronic tongue system to verify their umami intensity and flavor attributes. Finally, molecular docking and 200 ns molecular dynamics (MD) simulations were performed to elucidate the molecular mechanisms governing the interactions between these peptides and the T1R1/T1R3 receptor. By analyzing the conformational stability and residue flexibility of the receptor, this research aims to provide a robust theoretical and practical framework for the high-throughput discovery and mechanistic exploration of food-derived flavor peptides.

## 2 Materials and methods

### 2.1 Materials

The four targeted peptides, ELLADMK, HELDTK, ADVLEK, and LEELMDK, with a purity of ≥ 95%, were synthesized by Niu Ji Biotechnology Co., Ltd. (Liaoning, China).

### 2.2 *In silico* digestion of the sunflower proteome

The proteome sequences of sunflower (*Helianthus annuus* L.) were retrieved from the NCBI protein database (https://static.pubmed.gov/portal/portal.fcgi/, accessed on 10 January 2026). A custom Python script was employed to perform in silico complete trypsin digestion of the protein sequences. The cleavage sites were specifically defined at the carboxyl terminus of Lys (K) and Arg (R), excluding the Lys-Pro and Arg-Pro bonds. This virtual hydrolysis generated an initial library of 1,828,570 peptide fragments. After deduplication, 957,995 unique sequences were retained. Subsequently, sequences consisting of 4 to 8 amino acid residues were filtered, resulting in a targeted short peptide database comprising 265,051 sequences.

### 2.3 Machine learning model construction and evaluation

A dataset of umami peptides was downloaded from the TastePeptides-Meta (19) database (http://tastepeptides-meta.com/TastePeptidesDB, accessed on 12 January 2026). Verified umami peptides were designated as positive samples, while non-umami peptides served as negative samples. Several physicochemical properties were calculated for the positive samples, including molecular weight (MW), isoelectric point (pI), net charge at pH 7.0, grand average of hydropathy (GRAVY), and amino acid composition. These properties were utilized to conduct a preliminary screening of the sunflower short peptide database.

To computationally represent the peptide sequences, the pre-trained protein language model ESM-2 (specifically, the esm2_t33_650M_UR50D architecture) was employed to extract high-dimensional feature embeddings for both positive and negative samples. Utilizing these ESM-2 derived feature vectors as inputs, four machine learning models were trained for the binary classification task: Extreme Gradient Boosting (XGBoost), Light Gradient Boosting Machine (LightGBM), Multilayer Perceptron (MLP), and Transformer. The predictive performance of these models was comprehensively evaluated using the following metrics: accuracy (ACC), area under the receiver operating characteristic curve (AUC), Matthews correlation coefficient (MCC), precision (PREC), recall (REC), and F1 score. Based on the overall performance on the test set, the optimal model was selected for the subsequent virtual screening.

### 2.4 Peptide screening and electronic tongue evaluation

The optimized LightGBM model was employed to predict and score the short peptides within the sunflower database. The top four ranked sequences (ELLADMK, HELDTK, ADVLEK, and LEELMDK) were selected as candidate umami peptides for further experimental validation.

The sensory profiles of these candidates were characterized using an SA402B electronic tongue system (Insent Inc., Japan). All peptide samples were dissolved in deionized water to achieve a final mass concentration of 0.2 mg/mL and were thoroughly homogenized before testing. The measurement protocol was strictly standardized as follows: (1) Sensors were first rinsed in a cleaning solution for 90 s, followed by two consecutive stabilization steps in the first and second reference solutions for 120 s each, and were finally zeroed at the equilibrium position for 30 s. (2) The sensors were then immersed in the peptide sample solution to record the initial taste response for 30 s. After a rapid 3 s rinse in a post-reference solution, the sensors were immersed in a fresh reference solution for an additional 30 s to capture the aftertaste (change in membrane potential caused by adsorption, CPA) response.

### 2.5 Molecular docking

The umami taste receptor T1R1/T1R3 functions as a heterodimeric complex. The protein sequences for human T1R1 (TAS1R1, UniProt ID: Q7RTX1) and T1R3 (TAS1R3, UniProt ID: Q7RTX0) were obtained from the UniProt database. The three-dimensional structure of the T1R1/T1R3 heterodimer, predicted by AlphaFold3^1^, was utilized as the receptor model.

Blind docking was performed between the receptor and each of the four candidate sunflower peptides using the HPEPDOCK (20) web server.^2^ The entire protein structure was set as the target without predefining the binding sites. Among the multiple generated docking conformations for each peptide, the complex with the highest docking score was selected for subsequent structural analysis. The binding modes were visualized and analyzed using PyMOL 2.5 software.

### 2.6 Molecular dynamics simulation and trajectory analysis

The complexes obtained from molecular docking were used as the initial structures for MD simulations. The tLeap module in the AMBER software package was used to generate the force field parameters. The ff19SB force field was applied to the proteins, and the gaff2 force field was assigned to the ligands (21). All systems were solvated with the OPC water model in a cubic box, maintaining a minimum distance of 10 Å between the solute surface and the box edges . Periodic boundary conditions were applied to all five systems (including the apo receptor and the four peptide-receptor complexes) to eliminate edge effects, and an appropriate number of Na^+^ counterions were added to neutralize the systems. The SHAKE algorithm was employed to constrain all bonds involving hydrogen atoms. Non-bonded electrostatic interactions were calculated using the Particle Mesh Ewald (PME) method with a cutoff distance of 8 Å (22).

The energy minimization process consisted of 5,000 steps of the steepest descent method followed by 20,000 steps of the conjugate gradient method (23). The systems were then gradually heated to 300 K under the NVT ensemble, followed by a 50 ns equilibration simulation under the NPT ensemble. Once the systems reached equilibrium, a 200 ns production simulation was performed for each system using the pmemd.cuda module in AMBER 24, with a time step of 2 fs.

Post-processing of the production trajectories was conducted using the CPPTRAJ module in AMBER (24). Using the initial structure as a reference, the root-mean-square deviation (RMSD) (25) of the protein backbone Cα atoms and the radius of gyration (Rg) were calculated. All resulting plots and statistical analyses were generated using the MD_quick_plot toolkit (“MD_quick_plot: A Tool for Streamlined MD Trajectory Analysis,” 2025).

### 2.7 Statistical analysis

The experimental data were expressed as the mean ± standard deviation of three parallel sets of experiments. The significance level for statistical analysis was set at *p* < 0.05.

## 3 Results

### 3.1 Construction of the sunflower peptide database

A total of 75,694 protein sequences of sunflower (*Helianthus annuus* L.) were retrieved from the NCBI protein database. Following in silico trypsin digestion, 1,828,570 peptide fragments were generated. After deduplication, 957,995 unique peptide sequences were obtained.

Given that dipeptides and tripeptides typically exhibit weak umami intensity, whereas peptides exceeding eight amino acid residues are more prone to introducing bitterness or other off-flavors, sequences ranging from 4 to 8 amino acids in length were specifically targeted (26). Ultimately, 265,051 candidate short peptide sequences were retained, constituting the finalized sunflower peptide database for subsequent downstream screening.

### 3.2 Physicochemical property analysis of umami peptides

To establish effective in silico screening criteria, the physicochemical properties of reported umami peptides from the positive dataset were systematically analyzed (Figure 1). As illustrated in Figure 1A, the molecular weight (MW) of known umami peptides was predominantly concentrated in the range of 0–1,000 Da, exhibiting a distinct unimodal distribution. This indicates that umami peptides are primarily small-molecule oligopeptides. The isoelectric point (pI) mainly ranged from 4 to 7, spanning from weakly acidic to near-neutral (Figure 1B). Under physiological conditions (pH 7.0), the net charge was largely distributed between −4 and +4 with a relatively symmetrical pattern (Figure 1C), suggesting that umami peptides tend to be near electroneutral or carry weak charges in physiological environments. Furthermore, the grand average of hydropathicity (GRAVY) values were primarily clustered between −2 and 0 (Figure 1D), revealing a strong hydrophilic nature for most umami peptides. This characteristic is likely closely associated with their excellent aqueous solubility and subsequent taste-active properties. The amino acid composition analysis (Figure 1E) further unveiled that glutamic acid (Glu, E) accounted for the highest average proportion (14.14%) in umami peptides, followed by aspartic acid (Asp., D) (9.01%) and leucine (Leu, L) (8.46%). These findings underscore the critical role of acidic and moderately hydrophobic amino acids in the structural formation and taste manifestation of umami peptides.

**Figure 1:**
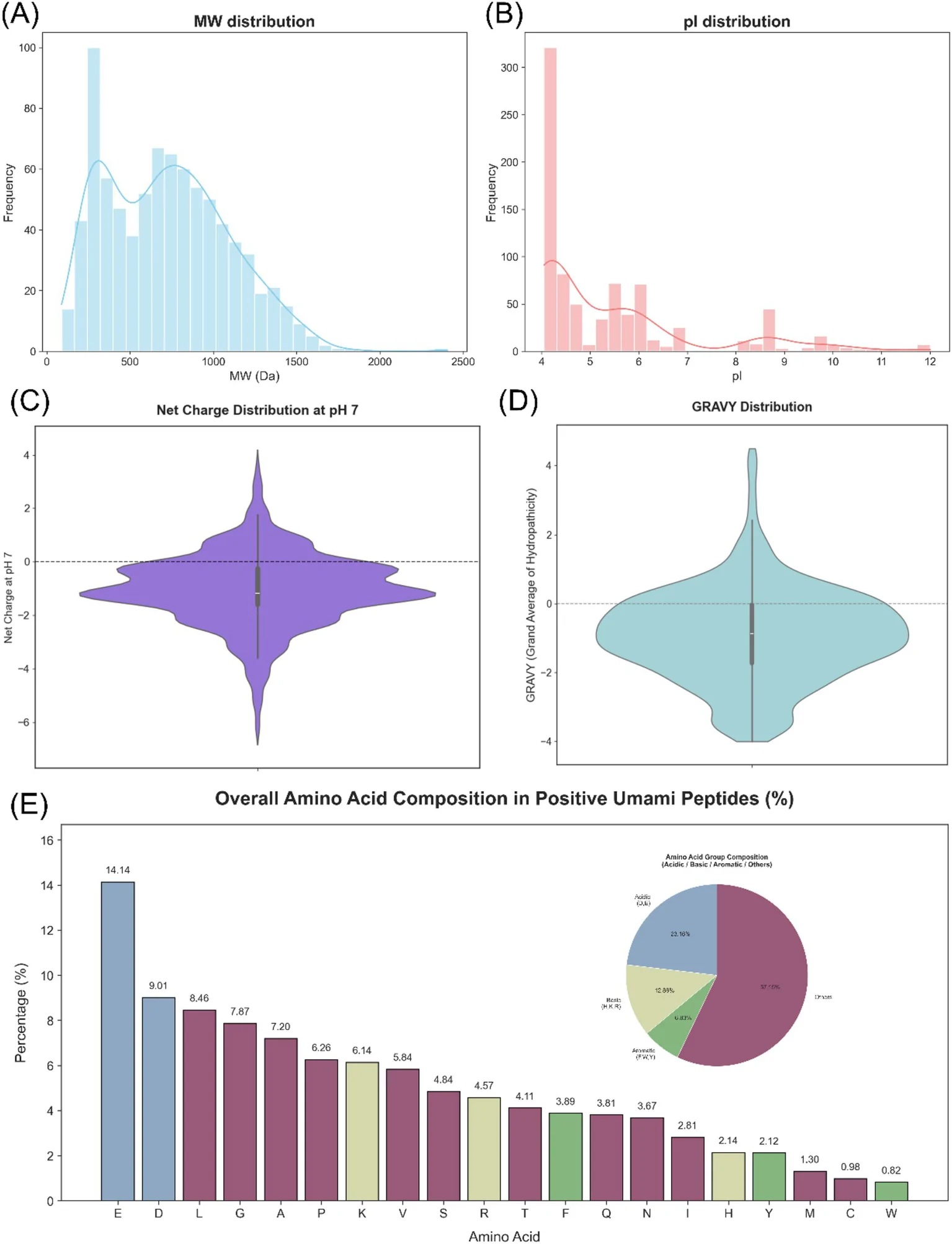
Comprehensive physicochemical property profiles of verified umami peptides from the positive dataset. **(A)** Molecular weight (MW) distribution, demonstrating a strong preference for low-molecular-weight oligopeptides. **(B)** Isoelectric point (pI) distribution, indicating a predominantly acidic nature across the dataset. **(C)** Violin plot of net charge distribution at physiological pH (7.0), highlighting an overall negative charge profile. **(D)** Violin plot of the grand average of hydropathicity (GRAVY) distribution, confirming the highly hydrophilic characteristics of these peptides. **(E)** Overall amino acid composition (bar chart) and the relative proportion of functional amino acid groups (inset pie chart: acidic, basic, aromatic, and others).

Based on these comprehensive profiles, preliminary filtering rules were established as follows: MW < 1,000 Da; acidic pI ranging from 3.0 to 6.5; negative net charge at pH 7.0 ranging from −3.5 to 0; GRAVY ≤ 0; proportion of acidic amino acids (Asp + Glu) ≥ 25%; and proportion of aromatic amino acids (Phe + Trp + Tyr) ≤ 25%. By applying these empirical criteria, 50,079 potential umami peptide candidates were successfully screened from the initial database of 265,051 short peptides.

### 3.3 Performance evaluation of machine learning models and identification of umami candidates

To accurately identify potential umami sequences from the filtered candidates, four machine learning models, including XGBoost, LightGBM, MLP, and Transformer, were trained for binary classification using the positive and negative sample datasets. Their predictive performances were comparatively evaluated using five-fold cross-validation based on multiple performance metrics, ROC curves, and out-of-fold confusion matrices (Figures 2, 3; Table 1).

**Figure 2:**
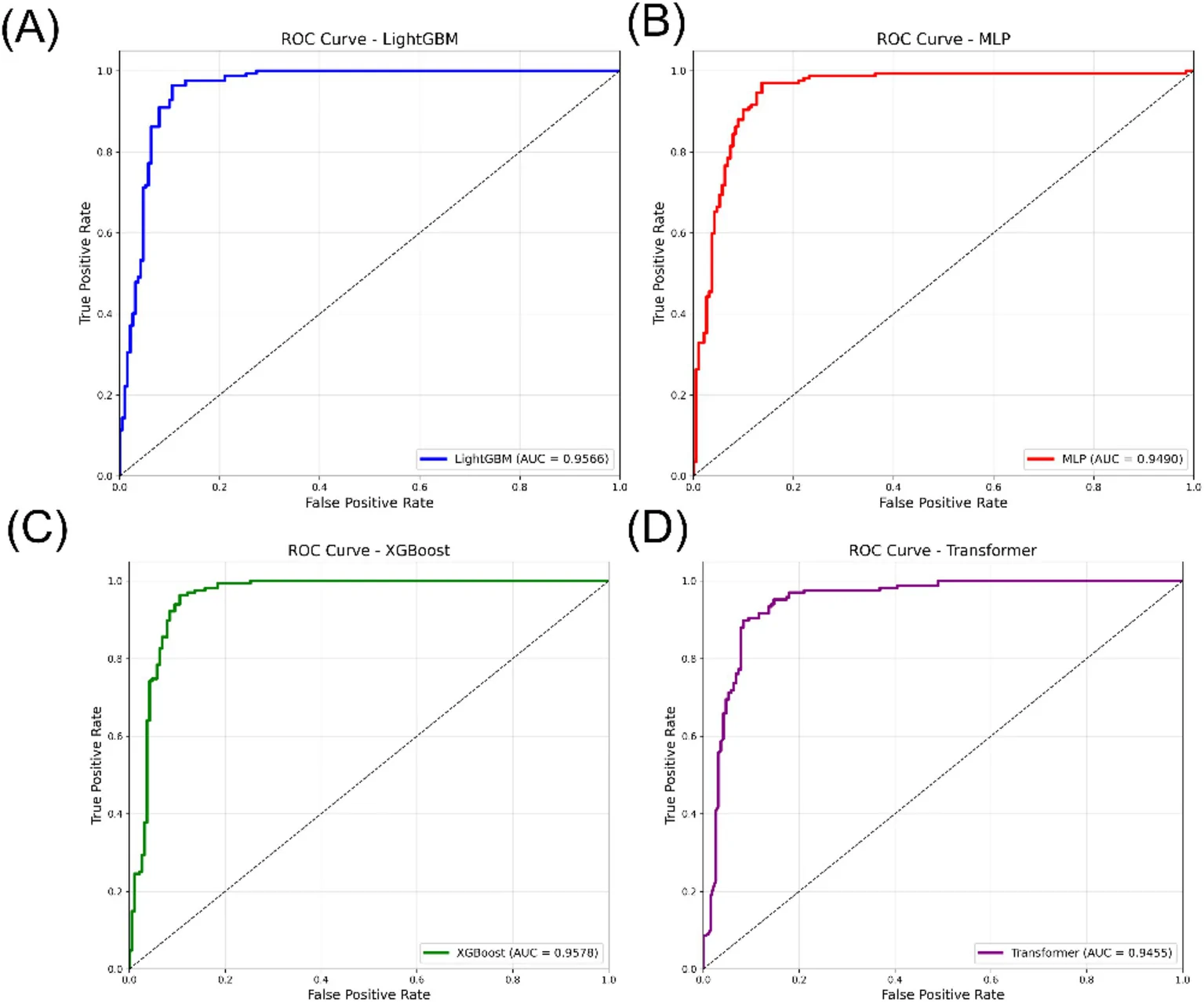
Receiver operating characteristic (ROC) curves of the four evaluated machine learning models for umami peptide classification based on ESM-2 sequence feature embeddings. **(A)** LightGBM, which exhibited the most stable and robust discriminatory capacity across varying thresholds; **(B)** Multilayer Perceptron (MLP); **(C)** XGBoost, showing highly comparable performance to LightGBM; and **(D)** Transformer, which displayed a higher false-positive rate.

**Figure 3:**
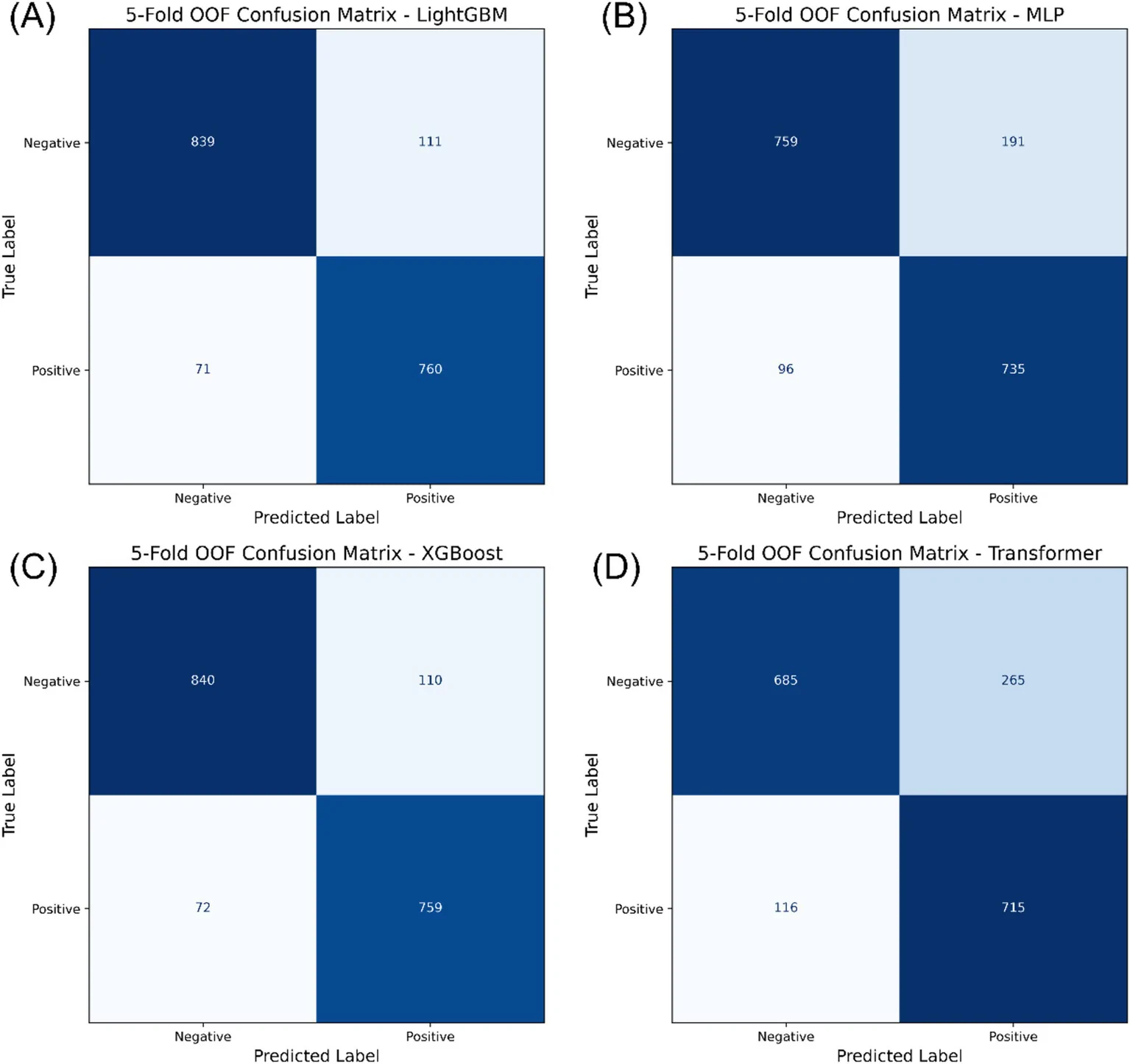
Out-of-fold confusion matrices evaluating the binary classification performance of the four machine learning models. **(A)** LightGBM, **(B)** Multilayer Perceptron (MLP), **(C)** XGBoost, and **(D)** Transformer. The matrices visually detail the absolute counts of true-positive, true-negative, false-positive, and false-negative predictions.

**Table 1:** Comprehensive performance evaluation metrics of the four machine learning models (LightGBM, XGBoost, MLP, and Transformer) for umami peptide binary classification.

Model	ACC	AUC	MCC	PREC	REC	F1
LightGBM	0.8984 ± 0.0041	0.9480 ± 0.0111	0.7972 ± 0.0074	0.8976 ± 0.0117	0.9121 ± 0.0092	0.8934 ± 0.0034
XGBoost	0.8956 ± 0.0051	0.9506 ± 0.0098	0.7914 ± 0.0101	0.8739 ± 0.0093	0.9073 ± 0.0131	0.8902 ± 0.0055
MLP	0.8938 ± 0.0166	0.9296 ± 0.0119	0.7665 ± 0.0335	0.8826 ± 0.0210	0.8665 ± 0.0170	0.8744 ± 0.0176
Transformer	0.8770 ± 0.0111	0.9318 ± 0.0149	0.7531 ± 0.0223	0.8875 ± 0.0153	0.8436 ± 0.0107	0.8649 ± 0.0117

The table details Accuracy (ACC), Area Under the Curve (AUC), Matthews Correlation Coefficient (MCC), Precision, Recall, and F1 score for each model based on five-fold cross-validation. The aggregated metrics quantitatively demonstrate that LightGBM achieves the most optimal balance between precision and recall (highest F1 and MCC), solidifying its selection as the core predictive model for the virtual screening pipeline. ACC, accuracy; AUC, area under the receiver operating characteristic curve; MCC, Matthews correlation coefficient; PREC, precision; REC, recall; F1, F1 score. All values are expressed as the mean ± standard deviation.

Among the four models, LightGBM exhibited the best overall and most balanced performance. It achieved an accuracy of 0.8984 ± 0.0041, an AUC of 0.9480 ± 0.0111, and an MCC of 0.7972 ± 0.0074. The ROC curve for LightGBM (Figure 2A) displayed a steep initial ascent and remained highly stable, indicating an excellent true-positive rate even at low false-positive thresholds, which confirms its robust discriminatory capacity across varying classification cutoffs. The out-of-fold confusion matrix further showed that LightGBM correctly classified 839 negative and 760 positive samples, while producing only 111 false-positive and 71 false-negative predictions (Figure 3A). This exceptionally low false-positive rate is practically crucial for virtual screening pipelines, as it minimizes the erroneous selection of non-umami peptides, thereby significantly reducing downstream experimental validation costs.

XGBoost showed a highly comparable classification performance and achieved a slightly higher AUC of 0.9506 ± 0.0098. Its confusion matrix contained 840 true-negative, 759 true-positive, 110 false-positive, and 72 false-negative predictions (Figure 3C), demonstrating a classification pattern almost identical to that of LightGBM. Nevertheless, considering the overall combination of accuracy, MCC, F1 score, and classification balance, LightGBM was selected as the core model.

The superior performance of LightGBM is fundamentally driven by its underlying algorithmic architecture. As a highly optimized gradient boosting framework, LightGBM employs a leaf-wise (best-first) tree growth strategy, which minimizes classification loss more aggressively and efficiently than traditional depth-wise growth. Moreover, LightGBM is exceptionally adept at handling dense, high-dimensional representations, allowing it to effectively capture the complex, non-linear relationships embedded within the pre-extracted ESM-2 sequence features. Deep learning architectures, such as MLP and Transformer, typically require massive datasets to optimize their extensive parameters; on this specific dataset scale, the Transformer model likely suffered from overfitting, generating the highest numbers of false predictions (265 FP, 116 FN). LightGBM successfully circumvented this overfitting limitation, maintaining a rigorous balance between precision and recall. While XGBoost showed comparable performance with a slightly higher AUC (0.9506 ± 0.0098), LightGBM’s optimal combination of MCC, F1 score (0.8934 ± 0.0034), and superior false-negative control ultimately justified its selection as the core model for subsequent high-throughput screening.

In comparison, the MLP model showed a moderate reduction in classification performance, with 759 true-negative and 735 true-positive predictions, accompanied by 191 false-positive and 96 false-negative predictions (Figure 3B). The Transformer model exhibited the weakest overall performance, generating the largest numbers of false-positive and false-negative predictions, at 265 and 116, respectively (Figure 3D). In particular, its relatively high false-positive rate indicated a reduced ability to distinguish non-umami peptides from umami peptides. Taken together, the metric-based evaluation and confusion-matrix analysis consistently supported the selection of LightGBM for subsequent high-throughput virtual screening of potential umami peptides.

The trained LightGBM model was subsequently utilized to predict the umami probability scores of the 50,079 candidate sequences. The sequences were ranked based on their predictive scores, and the top 10 potential umami peptides with the highest confidence were identified (Table 2). From this top-tier list, the four sequences with the highest predicted scores were selected for chemical synthesis and further experimental validation: ELLADMK, HELDTK, ADVLEK, and LEELMDK.

**Table 2:** The top 10 potential sunflower-derived umami peptides identified by the optimal LightGBM model, ranked by their predictive probability scores.

Sequence	Umami_Probability
ELLADMK	0.993660343
HELDTK	0.992956117
ADVLEK	0.992764676
LEELMDK	0.992439383
VEEGHK	0.991988448
LSVDEK	0.991905481
TDLVEK	0.991791826
EELVTK	0.991716219
EAQLEVK	0.99171132

These candidates were efficiently computationally screened from the comprehensive virtual trypsin hydrolysis library of the *Helianthus annuus* L. proteome. Peptides exhibiting the highest machine learning confidence scores (including ELLADMK, HELDTK, ADVLEK, and LEELMDK) were subsequently selected for chemical synthesis and rigorous electronic tongue sensory validation.

### 3.4 Sensory evaluation of potential sunflower umami peptides via electronic tongue

The sensory characteristics of the four synthesized candidate sunflower peptides (HELDTK, ELLADMK, ADVLEK, and LEELMDK) were rigorously evaluated using an electronic tongue system. A multifaceted analysis (Figure 4) systematically deconstructs their sensory differentiation and flavor profiles.

**Figure 4:**
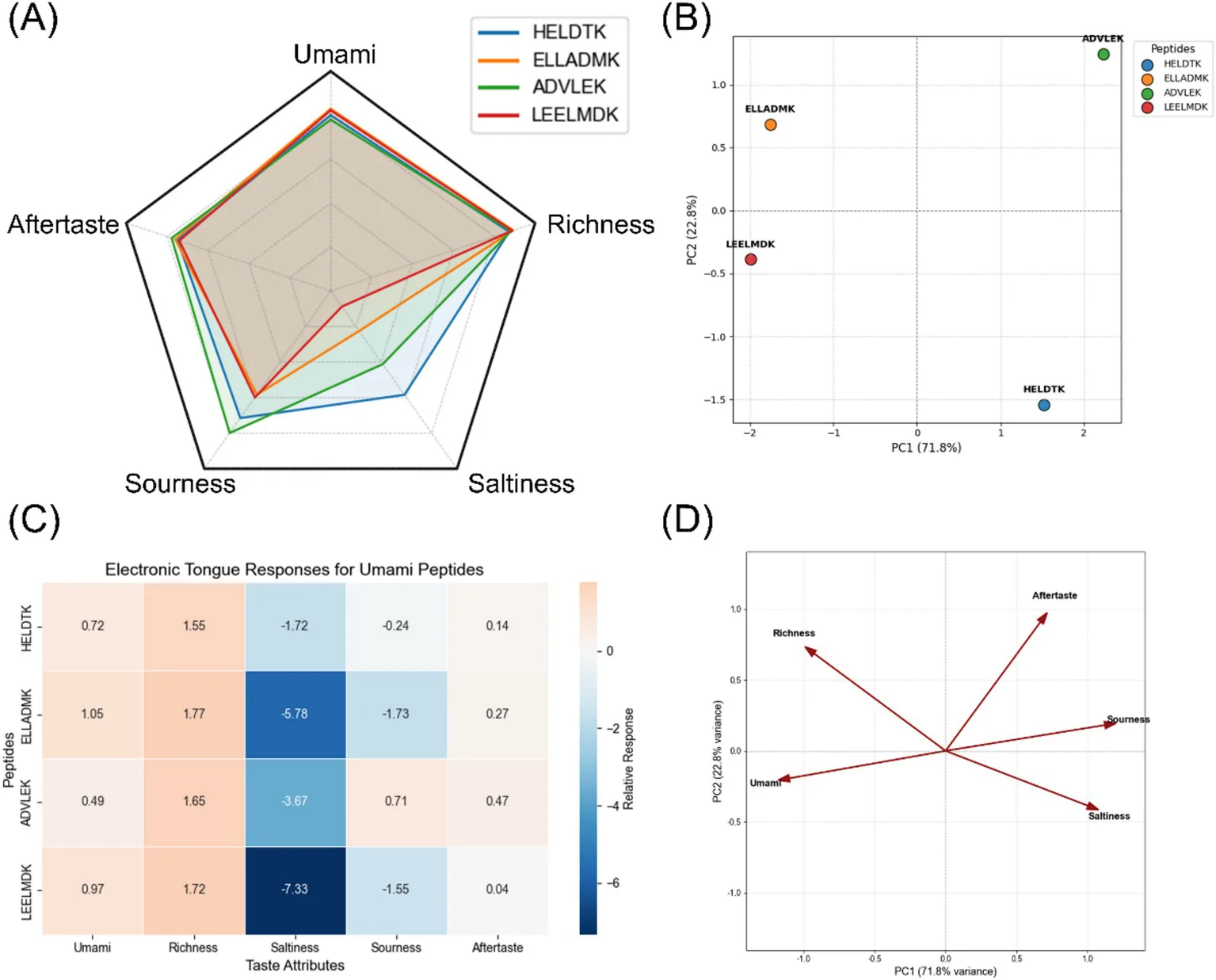
Comprehensive sensory evaluation and flavor profiling of the four candidate sunflower umami peptides (HELDTK, ELLADMK, ADVLEK, and LEELMDK) using an electronic tongue system. **(A)** Radar plots of the five primary taste attributes, visually demonstrating that umami and richness are the dominant flavor dimensions for all candidates. **(B)** Principal Component Analysis (PCA) score plot illustrating the spatial distribution of the peptide samples, effectively discriminating the high-intensity sensory profiles of ELLADMK and LEELMDK (far-left clustering) from the comparatively weaker profiles of ADVLEK and HELDTK. **(C)** Heatmap of quantitative electronic tongue relative responses, highlighting the specific high-intensity scores for umami and richness, alongside strong negative responses (sensory suppression) for undesirable tastes such as saltiness. **(D)** PCA loading plot elucidating the synergistic relationships between taste attributes, confirming that the umami and richness vectors are tightly correlated and drive the primary sensory differentiation (PC1).

The integration of radar plots (Figure 4A) and the quantitative heatmap (Figure 4C) reveals a distinct flavor architecture characterized by dominant umami and richness alongside significant taste suppression in other channels. For all candidate peptides, richness and umami constituted the primary positive sensory vectors. ELLADMK exhibited the most potent absolute responses, yielding the highest values in both umami (1.05) and richness (1.77) channels, closely followed by LEELMDK (umami: 0.97; richness: 1.72). Concurrently, the heatmap reveals pronounced negative responses for saltiness—particularly for LEELMDK (−7.33) and ELLADMK (−6.78)—and sourness, indicating that these highly active umami peptides effectively suppress undesirable flavor notes, resulting in a cleaner, more synergistic umami profile. In contrast, ADVLEK (umami: 0.49) and HELDTK (umami: 0.72) displayed markedly weaker positive sensory intensities and less pronounced suppression of secondary tastes.

Principal Component Analysis (PCA) successfully discriminated the sensory characteristics of the peptides, with the first two principal components capturing 93.8% of the total variance (PC1: 71.8%; PC2: 22.0%). The loading plot (Figure 4D) elucidates the internal correlations among taste attributes: the vectors for “umami” and “richness” project strongly along the negative axis of PC1, confirming their tight synergy in defining the primary flavor profile. Conversely, sourness and saltiness vectors align oppositely along the positive PC1 axis, acting as antagonistic features to the umami-richness cluster.

This vector geometry directly explains the spatial distribution of the peptides in the score plot (Figure 4B). ELLADMK and LEELMDK are distinctly clustered in the far-left quadrants (negative PC1), perfectly aligning with the umami and richness directional vectors and corroborating their superior, high-intensity flavor profiles. Conversely, ADVLEK and HELDTK are positioned in the right-hand quadrants (positive PC1), driven by their comparatively weaker umami responses and different secondary taste profiles. Overall, the integrated electronic tongue data systematically confirms that ELLADMK and LEELMDK possess the most desirable and structurally balanced umami characteristics among the candidates.

### 3.5 Molecular docking analysis

Molecular docking simulations were conducted to elucidate the detailed binding modes of the four candidate sunflower peptides (ELLADMK, HELDTK, ADVLEK, and LEELMDK) within the taste receptor T1R1/T1R3 complex. All four peptides were found to be effectively bound to the ligand-binding pocket within the Venus Flytrap Domain (VFD) region of the heterodimeric complex (Figures 5A,B). Their binding sites were primarily located within the VFD cleft, a region notably rich in polar and charged residues.

**Figure 5:**
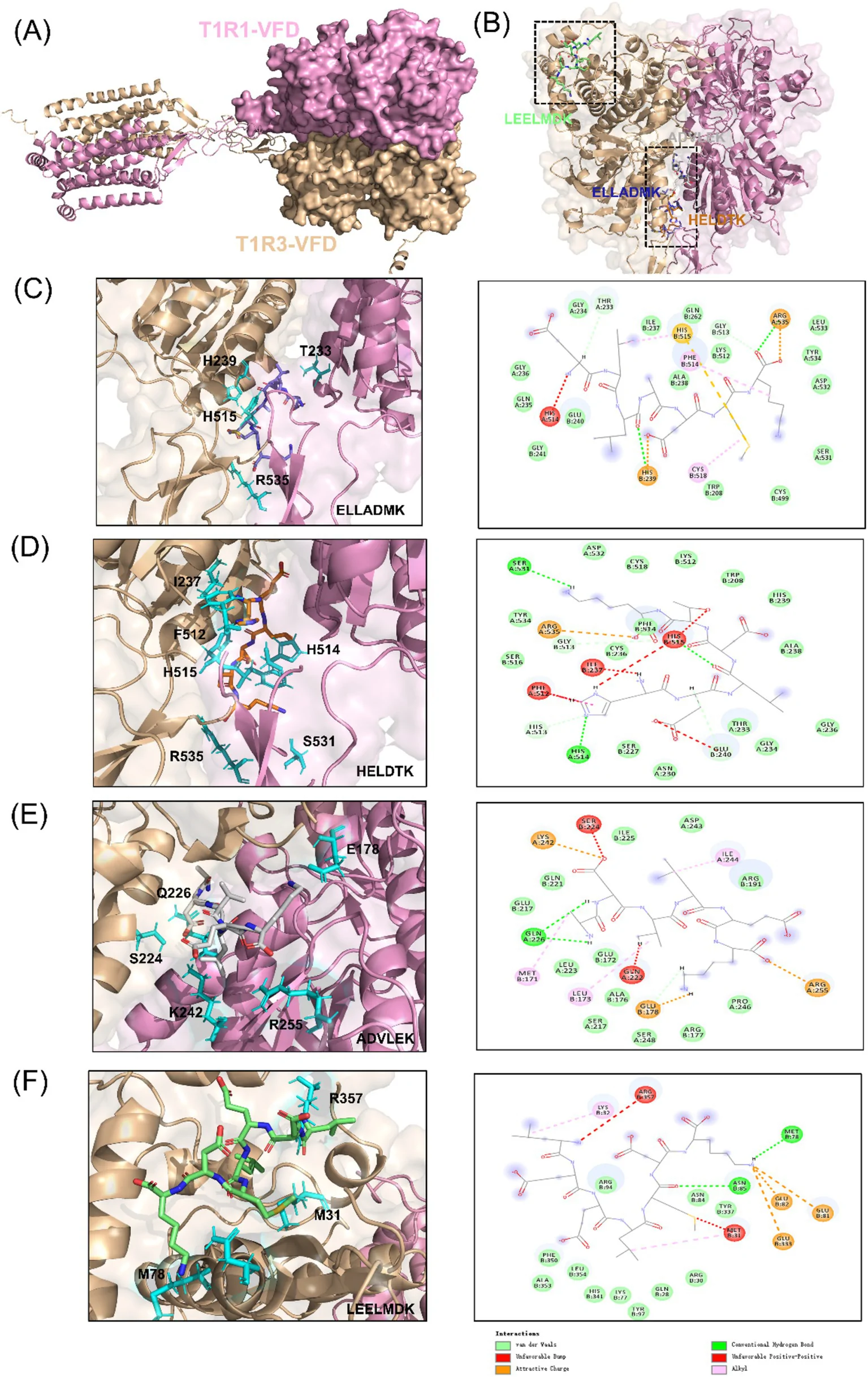
Molecular docking analysis elucidating the binding modes and structural interactions between the candidate sunflower umami peptides and the T1R1/T1R3 taste receptor. **(A)** Surface representation of the heterodimeric T1R1/T1R3 complex. **(B)** Overview of the ligand-binding pocket, demonstrating that all four peptides effectively anchor within the Venus flytrap domain (VFD) cleft, a region highly enriched in polar and charged residues. **(C–F)** Detailed 3D binding conformations and 2D interaction networks for ELLADMK, HELDTK, ADVLEK, and LEELMDK, respectively.

A detailed examination of specific interactions revealed unique binding profiles for each peptide, with the binding stabilized by a combination of hydrogen bonds and electrostatic interactions. For ELLADMK, key interactions involved hydrogen bonds and salt bridges with the T1R1 residues Arg151, His616, and Thr261 (Figure 5C). HELDTK was stabilized by a dense network of hydrogen bonds and hydrophobic contacts with His614, Phe165, Leu237, Arg656, and His651 (Figure 5D). The binding of ADVLEK was primarily driven by hydrogen bonds and salt bridges with Asp228, Glu373, His242, Glu224, and Arg356 (Figure 5E). For LEELMDK, interactions included hydrogen bonds, hydrophobic contacts, and π-alkyl interactions with His367, Met21, and Met78 (Figure 5F). The acidic residues of this peptide played a critical role in driving strong electrostatic attraction with the receptor complex. Overall, the results indicate that interactions between the acidic amino acids (Asp and Glu) of the peptides and the positively charged residues (e.g., arginine and histidine series) of the receptor are the key driving factor for binding. These findings are highly consistent with the umami taste activity assessment derived from the electronic tongue analysis, with ELLADMK and LEELMDK showing more extensive and stable hydrogen bond networks and higher binding potentials.

### 3.6 Structural stability and dynamic features of the receptor-peptide complexes

Through 200 ns molecular dynamics (MD) simulations, the structural stability and conformational changes of the umami receptor Venus flytrap domain (VFD) were comprehensively analyzed in its unbound (APO) state and when complexed with the four candidate umami peptides (ELLADMK, HELDTK, ADVLEK, and LEELMDK) (Figure 6).

**Figure 6:**
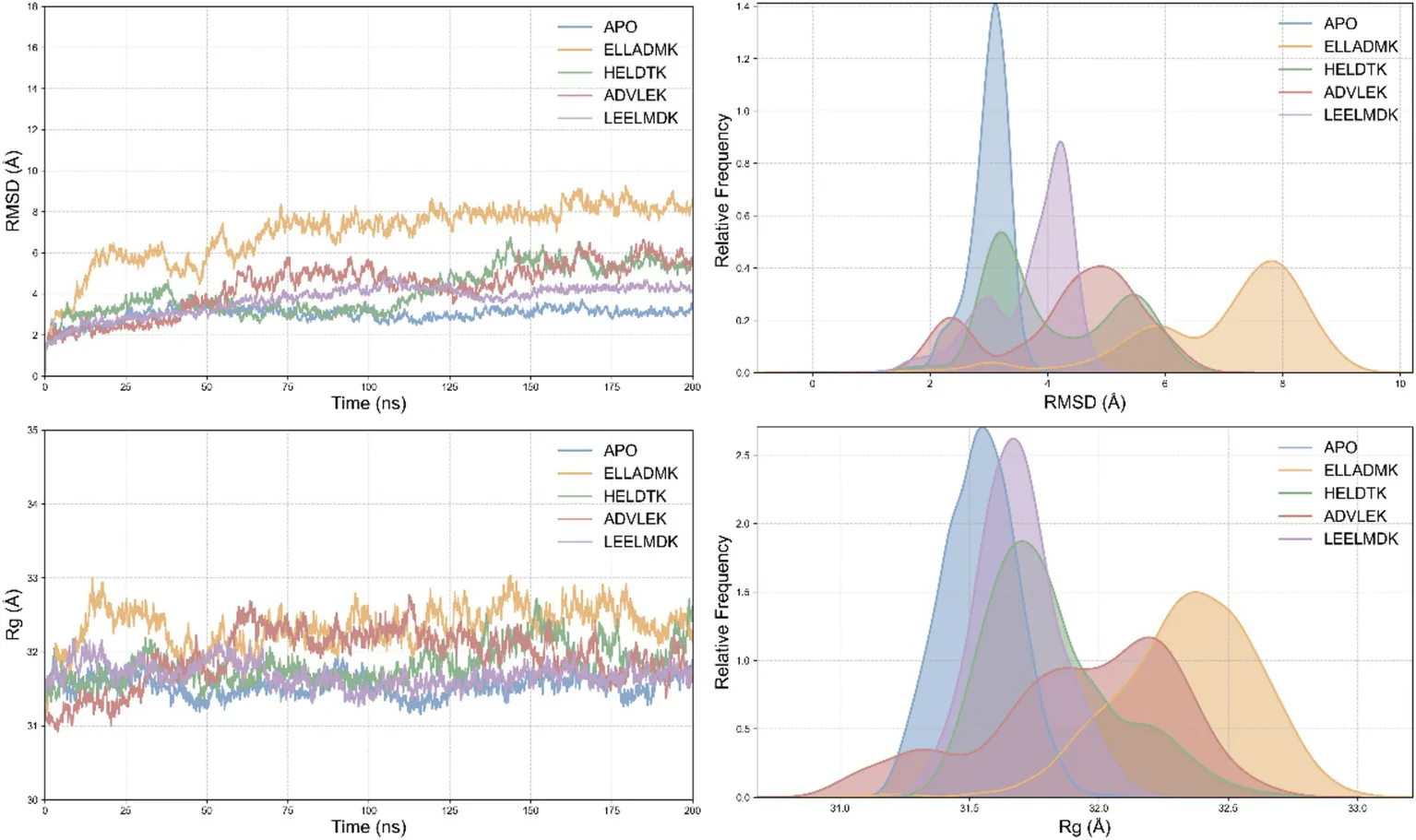
Structural stability and compactness dynamics of the T1R1/T1R3 Venus flytrap domain (VFD) in the unbound (APO) state and complexed with four candidate umami peptides over a 200 ns molecular dynamics (MD) simulation.

The Root Mean Square Deviation (RMSD) trajectories tracked the temporal evolution of structural stability throughout the simulation. The backbone conformation of the APO system was the most stable, rapidly reaching equilibrium within the first 20 ns and consistently maintaining a low RMSD value within the 2–4 Å range with minimal fluctuation. Conversely, peptide binding disrupted this inherent stability, triggering distinct dynamic adaptation phases. Notably, the ELLADMK-bound system underwent a prolonged conformational transition: its RMSD exhibited a steep continuous ascent during the initial 60 ns, followed by high-amplitude fluctuations, before finally converging to an elevated state of 8–10 Å in the latter half of the simulation. This intensive dynamic process indicates a profound and continuous receptor rearrangement. The systems bound to LEELMDK, HELDTK, and ADVLEK displayed moderate destabilization, overcoming initial structural adjustments to stabilize in the 4–8 Å range after approximately 40–50 ns.

The Radius of Gyration (R_g_) analysis further elucidated the continuous temporal process of structural expansion. The APO system maintained a highly compact and invariant conformation throughout the entire 200 ns, with R_g_ values strictly stabilizing between 31.0 and 31.5 Å. Upon peptide binding, the VFD underwent progressive spatial relaxation. Specifically, the binding of ELLADMK triggered the most substantial and continuous structural expansion; its R_g_ trajectory steadily climbed during the initial simulation phase, eventually plateauing at 32.0–32.5 Å. The other three peptides induced intermediate and more rapid R_g_ equilibrations. These continuous temporal trends were robustly validated by the relative frequency distribution plots of RMSD and R_g_, where the sharp, low-value peaks of the APO system contrasted sharply with the broad, right-shifted distribution peaks of the ELLADMK-bound system. Collectively, tracking this whole dynamic process demonstrates that umami peptides—particularly ELLADMK—induce a progressive, time-dependent conformational expansion and flexibility increase in the VFD, exerting a dramatic modulatory effect on receptor activation.

Solvent-accessible surface area analysis provided additional evidence for peptide-induced changes in receptor exposure (Figure 7). The SASA trajectories of all systems remained within relatively stable ranges over the 200 ns simulation, with no continuous upward or downward drift, indicating the absence of extensive structural collapse or unfolding. Nevertheless, peptide binding altered the average solvent exposure and distribution of the VFD. The APO system was generally distributed toward lower SASA values, whereas the peptide-bound systems showed varying degrees of rightward shift and distribution broadening. The ADVLEK- and LEELMDK-bound systems tended to occupy relatively higher SASA ranges, while ELLADMK and HELDTK produced intermediate shifts. These changes suggest that peptide binding modifies the accessibility of the receptor surface and induces local or global conformational rearrangements, although the SASA results do not indicate that ELLADMK produced the greatest increase in solvent exposure.

**Figure 7:**
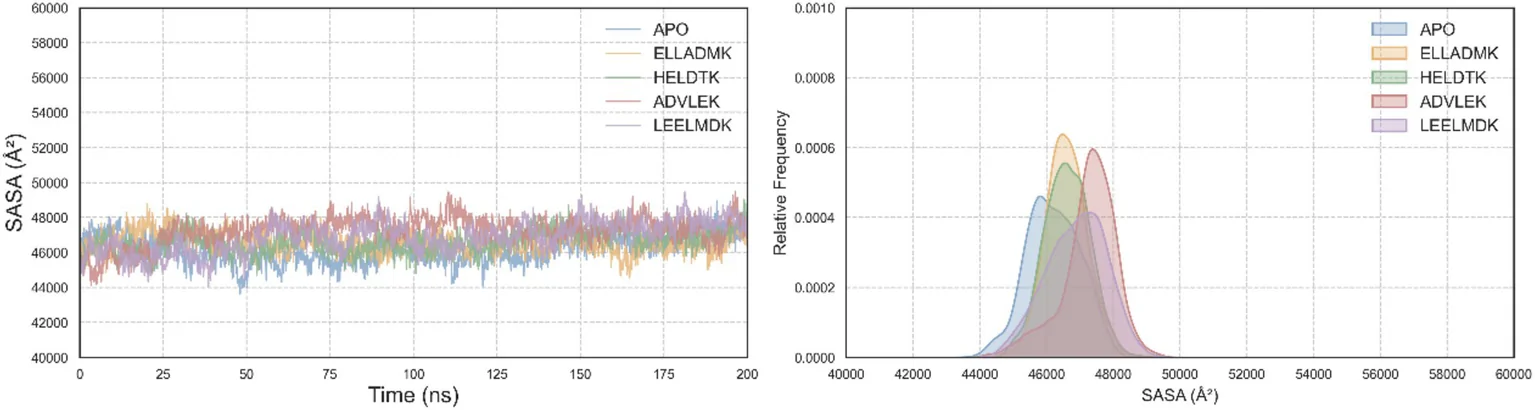
Solvent-accessible surface area (SASA) analysis of the T1R1/T1R3 receptor in the APO state and peptide-bound complexes throughout the 200 ns molecular dynamics simulations.

Taken together, the RMSD, Rg, and SASA analyses demonstrate that umami peptide binding alters the conformational stability, compactness, and solvent accessibility of the receptor VFD. These effects were peptide-dependent. ELLADMK induced the most pronounced changes in RMSD and Rg, whereas the SASA analysis revealed distinct but not fully identical patterns among the four peptide-bound systems. Therefore, the structural modulation caused by peptide binding involves multiple conformational properties rather than a uniform change across all dynamic indicators.

The Root Mean Square Fluctuation (RMSF) analysis (Figure 8) demonstrated that the binding of umami peptides significantly altered the residue flexibility of the receptor VFD. The APO system displayed the lowest overall residue flexibility. In contrast, the RMSF values for all peptide-bound systems increased, with ELLADMK and LEELMDK exerting the most prominent effects. Analysis of key local regions revealed specific flexibility variation patterns tightly linked to receptor function. The 460–510 region, which encompasses critical structural loops near the VFD binding pocket, exhibited notable spatial fluctuations upon peptide binding. This increased flexibility likely facilitates the dynamic closure and conformational rearrangement of the binding cleft necessary to physically secure the ligand. Furthermore, the flexibility of residues 705–755 increased significantly, particularly following ELLADMK binding. This region is critically positioned at the C-terminal base of the VFD, acting as a mechanical hinge that connects to the cysteine-rich domain and the downstream transmembrane region. The profound structural perturbation in this linker region, as highlighted by the ΔRMSF difference plot, strongly suggests that the ligand-induced conformational expansion within the VFD is mechanically transmitted through this specific axis. Collectively, these results indicate that highly active umami peptides, such as ELLADMK, initiate the receptor activation pathway by selectively amplifying the flexibility of the binding pocket loops (460–510) and the signal-transmitting linker region (705–755), providing a vital structural basis for downstream sensory signal transduction.

**Figure 8:**
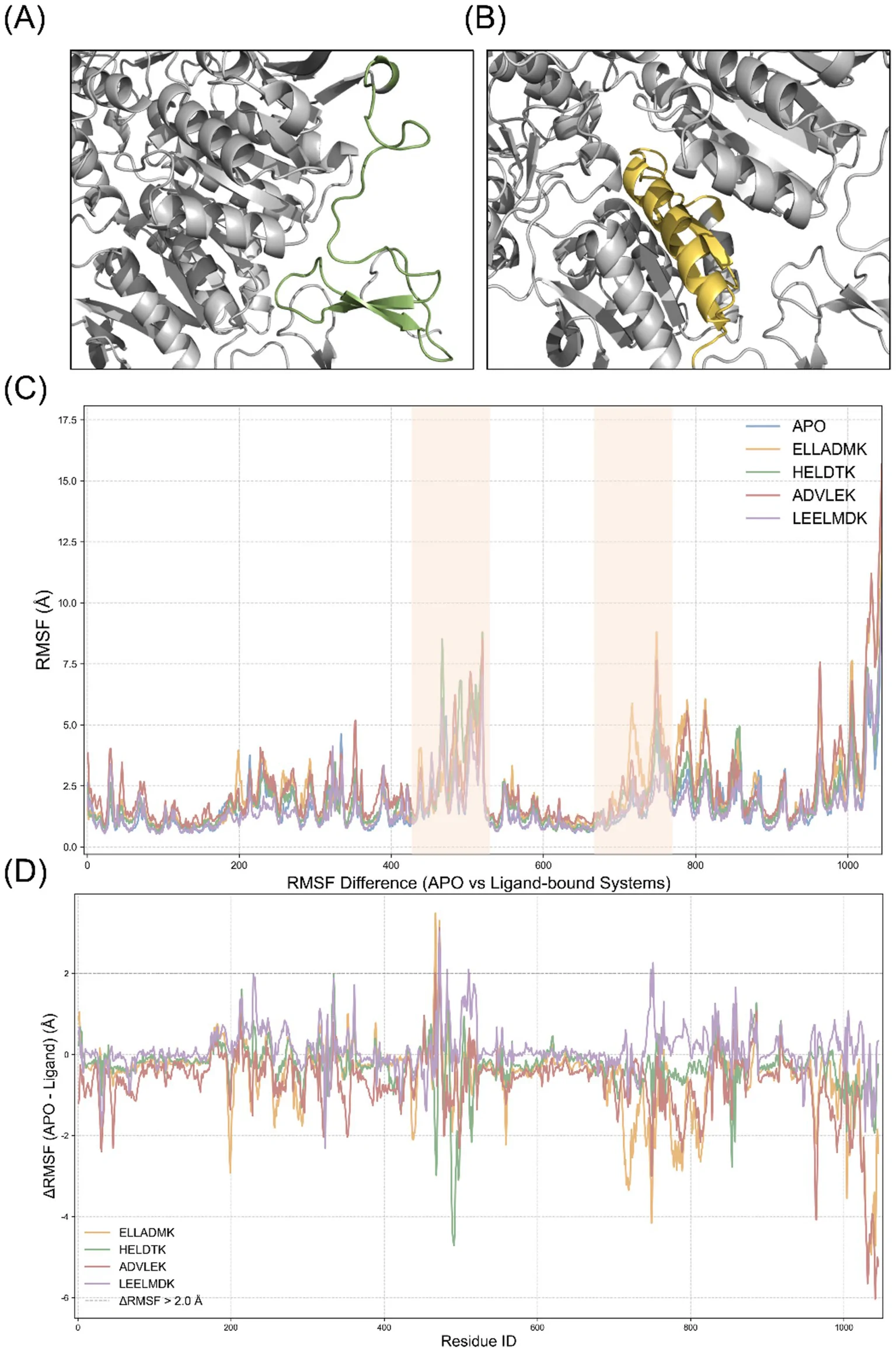
Comprehensive residue flexibility analysis of the T1R1/T1R3 Venus flytrap domain (VFD) before (APO state) and after peptide binding during the 200 ns molecular dynamics simulation. **(A,B)** 3D structural mapping of the critically flexible functional regions corresponding to residues 460–510 (green cartoon, **A**), which encompass essential structural loops near the ligand-binding pocket, and residues 705–755 (yellow cartoon, **B**), which act as a vital mechanical hinge connecting the VFD to downstream domains. **(C)** Root Mean Square Fluctuation (RMSF) profiles of the APO and the four peptide-bound systems, illustrating an overall increase in structural flexibility upon ligand binding compared to the rigid APO state. **(D)** The RMSF difference (ΔRMSF) between the APO and ligand-bound systems.

### 3.7 Secondary structure dynamics of the receptor

To comprehensively evaluate the conformational remodeling of the receptor, secondary structure dynamics were analyzed at both the global domain level (Supplementary Figure S1) and within localized binding regions (Figure 9). Global secondary structure evolution across the entire Venus flytrap domain (VFD) demonstrated that the core structural scaffold, primarily composed of rigid α-helices and β-sheets, remained largely intact across all systems throughout the simulation. This fundamental stability confirms that peptide binding does not induce catastrophic unfolding, but rather precise allosteric modulation. However, compared to the relatively static APO system, the peptide-bound complexes—particularly those involving ELLADMK and LEELMDK—exhibited elevated dynamic transitions across multiple non-core regions. These systems displayed an increased frequency of structural interconversions between unstructured coils, turns, and ordered elements across the broader VFD, indicating a state of global structural “breathing” and reorganization essential for mechanical signal propagation.

**Figure 9:**
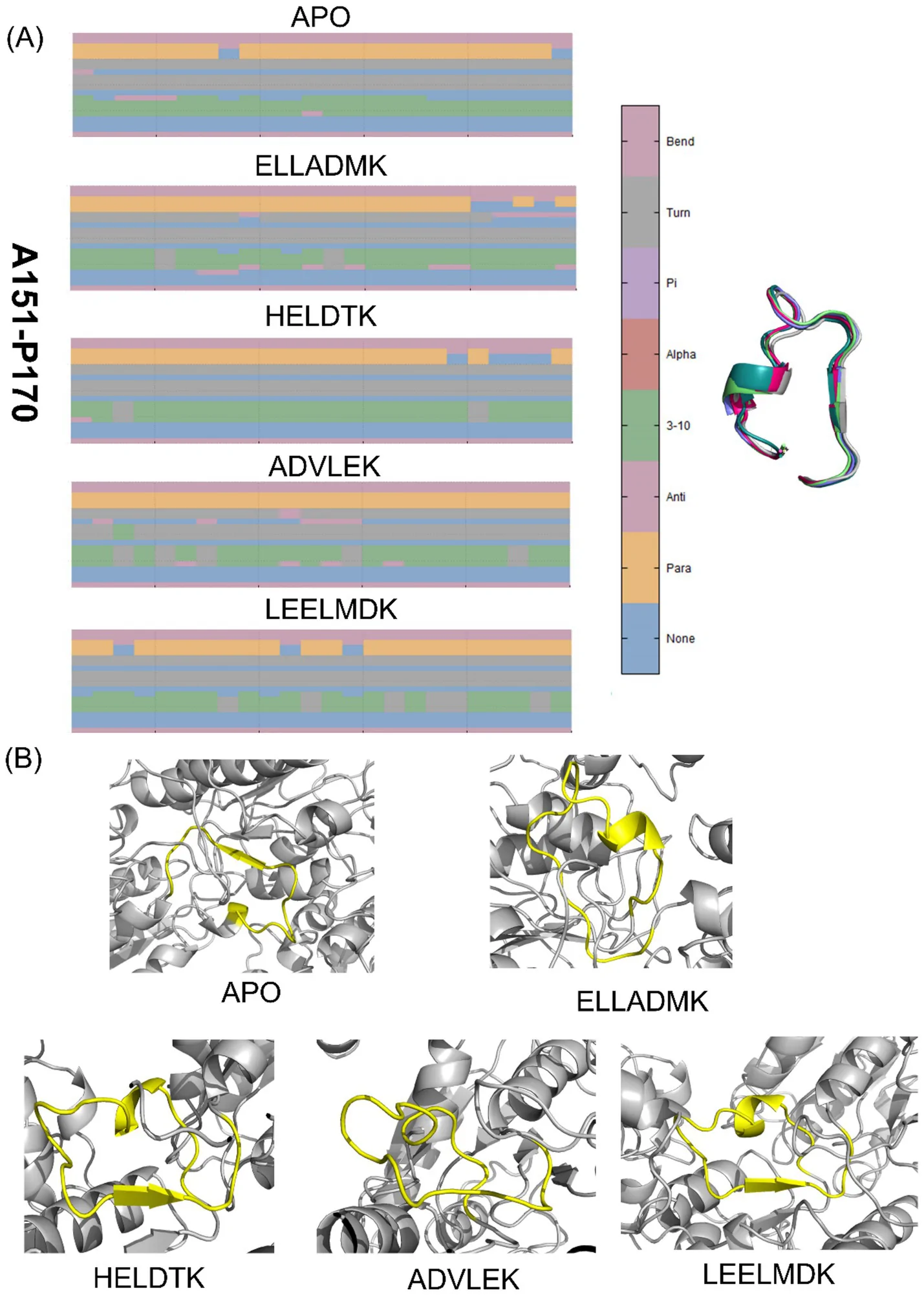
Secondary structure dynamics and localized conformational remodeling of the T1R1/T1R3 receptor focusing on the critical A151-P170 loop region near the ligand-binding pocket. **(A)** Time evolution of the secondary structure composition for residues 151–170 over the 200 ns molecular dynamics (MD) simulation across the APO state and the four peptide-bound systems. **(B)** Representative 3D structural snapshots highlighting the distinct localized conformational variations of the A151-P170 segment.

To further understand the localized conformational changes, the secondary structure dynamics of the receptor were analyzed (Figure 9). Dynamic analysis focusing on the region spanning residues 151–170 (A151-P170) revealed distinct conformational transitions induced by peptide binding. In the APO system, this specific region predominantly exhibited a random coil conformation (unstructured). However, the binding of the four umami peptides triggered highly specific secondary structure reorganizations.

Specifically, the binding of ELLADMK significantly increased the proportions of α-helices and β-bridges. Conversely, HELDTK binding substantially elevated the content of parallel β-sheets and uniquely induced the formation of π-helices. The presence of ADVLEK primarily promoted the generation of turn and bend structures. The structural modulations induced by LEELMDK within this region were intermediate, exhibiting characteristics bridging those of the APO and ELLADMK-bound states.

These findings highly suggest that distinct umami peptides can specifically modulate the secondary structure composition of this critical loop region. Such targeted structural adaptations likely drive the overall conformational rearrangement of the receptor VFD, playing a crucial role in the activation and initiation of the umami signal transduction pathway.

### 3.8 Principal component analysis and free energy landscape

To thoroughly explore the conformational space and thermodynamic stability of the T1R1/T1R3 receptor upon peptide binding, Principal Component Analysis (PCA) was performed on the MD trajectories, and the corresponding 3D Free Energy Landscapes (FEL) were constructed using the first two principal components (PCA1 and PCA2) (Figure 10). In these FEL plots, the deep red/orange basins represent the global minimum energy states, indicating the most thermodynamically stable conformations adopted by the protein during the simulation.

**Figure 10:**
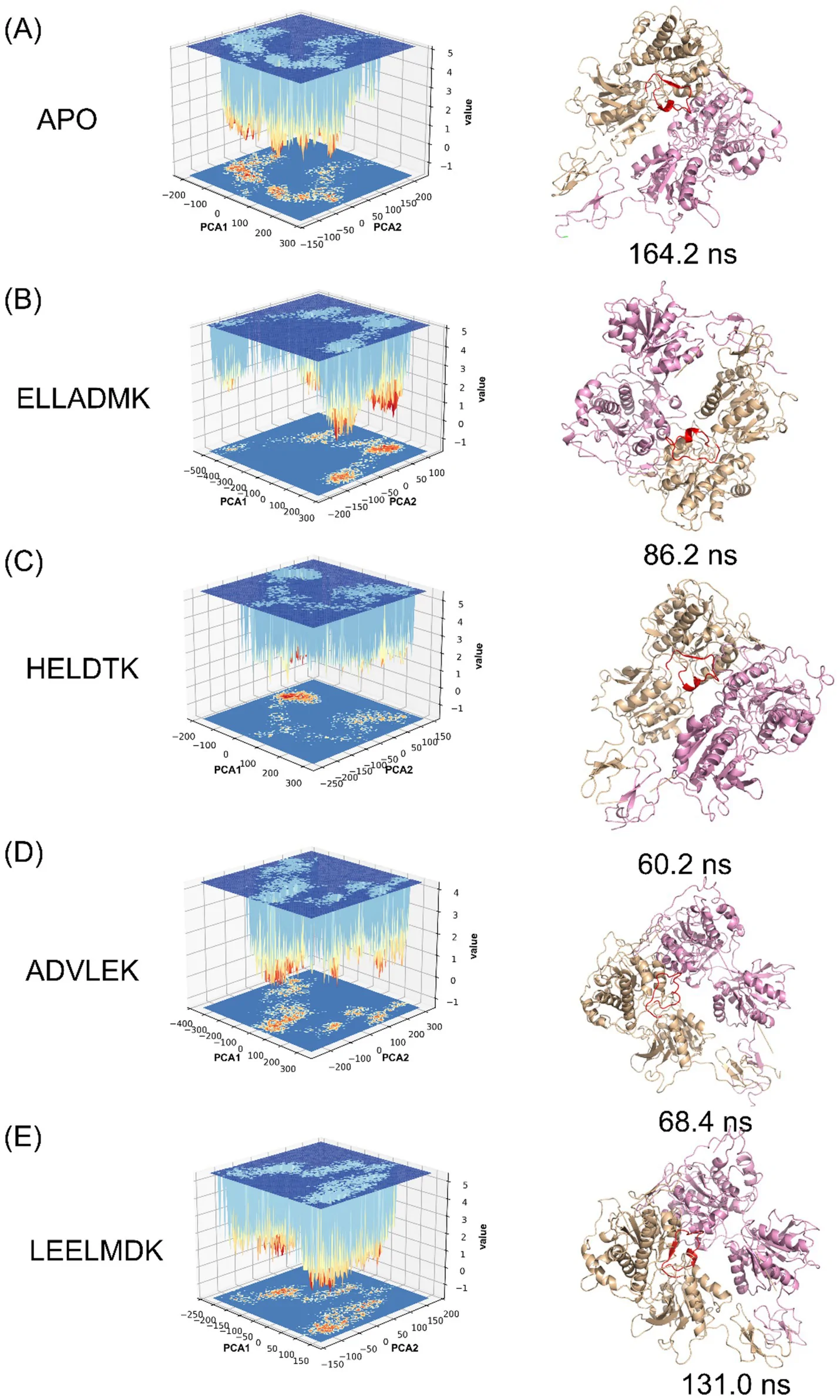
Principal component analysis (PCA) and 3D free energy landscapes (FEL) mapping the thermodynamic conformational space of the T1R1/T1R3 Venus flytrap domain (VFD). The 3D FEL maps are constructed for the **(A)** unbound APO, **(B)** ELLADMK, **(C)** HELDTK, **(D)** ADVLEK, and **(E)** LEELMDK systems using the first two principal components.

As illustrated in Figure 10A, the APO system exhibited a relatively broad and rugged conformational space with multiple scattered, shallow local energy minima. This suggests that the unbound receptor continuously fluctuates among several metastable states without locking into a single dominant conformation. However, the binding of the candidate umami peptides profoundly altered the free energy landscape, actively remodeling the receptor’s conformational subset in accordance with the induced-fit theory.

According to the induced-fit model, ligand binding thermodynamically drives the receptor to overcome internal energy barriers and adapt structurally to optimize complementarity. Notably, the binding of ELLADMK (Figure 10B) and LEELMDK (Figure 10E) induced a dramatic funneling of the landscape, resulting in highly concentrated, singular, and deep energy basins. This thermodynamic profile indicates that these two high-activity umami peptides possess sufficient binding energy to efficiently guide and firmly lock the receptor into a highly stable, thermodynamically favorable active conformation. The representative 3D structures extracted from these global minima highlight the specific, stabilized conformational rearrangement of the VFD cleft essential for robust signal transduction.

In contrast, the HELDTK (Figure 10C) and ADVLEK (Figure 10D) systems displayed broader or more scattered energy basins compared to ELLADMK. Viewed through the lens of induced-fit dynamics, these lower-activity peptides lack the optimal structural complementarity and energetic driving force required to fully overcome the receptor’s intrinsic energy barriers. Consequently, the receptor remains in a state of thermodynamic frustration, sampling multiple competitive, shallow substates rather than converging into a singular active conformation. This thermodynamic differentiation perfectly corroborates the electronic tongue sensory evaluation, where HELDTK and ADVLEK exhibited relatively weaker umami intensities.

Collectively, these FEL results demonstrate that umami efficacy is directly coupled to the peptide’s ability to execute a complete induced-fit transition. Highly potent umami peptides do not merely bind; they act as thermodynamic sculptors, driving the dynamic Venus flytrap domain into a specific, deep energy well that structurally triggers receptor activation.

### 3.9 Quantitative binding free energy analysis via MM/PBSA

Thermodynamic profiling of the peptide-receptor complexes was conducted using the MM/PBSA method to quantify the total binding free energies (ΔG_total_) and their constituent driving forces. The energetic breakdown reveals distinct interaction strategies among the candidate peptides. HELDTK exhibited the most thermodynamically stable binding profile with the lowest total binding free energy (−10.6800 ± 1.3828 kcal/mol). This affinity is heavily driven by a highly favorable electrostatic contribution (ΔE_ele_ = −191.0526 ± 4.4944 kcal/mol) that effectively overcomes its structural desolvation penalty. ADVLEK demonstrated a similarly electrostatically driven binding mode, yielding a favorable ΔG_total_ of −5.6505 ± 0.8874 kcal/mol.

Conversely, ELLADMK was characterized by the strongest van der Waals interactions (ΔE_vdw_ = −51.2040 ± 0.9871 kcal/mol) among all tested peptides, indicating superior geometric shape complementarity and dense hydrophobic packing within the receptor cleft. LEELMDK generated the most intense electrostatic attractions (ΔE_ele_ = −228.7382 ± 4.1515 kcal/mol); however, the massive polar solvation penalty (ΔG_solv_ = 283.3881 ± 3.8155 kcal/mol) associated with burying its highly charged residues resulted in a net positive binding free energy. These quantitative results suggest that while HELDTK and ADVLEK achieve the highest baseline thermodynamic stability, the strong van der Waals anchoring uniquely observed in ELLADMK may be the critical mechanical driver for the pronounced Venus flytrap domain (VFD) conformational expansion necessary for intense umami signal transduction (Table 3).

**Table 3:** Quantitative binding free energy analysis of the four candidate sunflower umami peptide-receptor complexes calculated using the MM/PBSA method.

System	ELLADMK	HELDTK	ADVLEK	LEELMDK
ΔE_vdw_	−51.2040 ± 0.9871	−28.7420 ± 0.6302	−30.8305 ± 0.7048	−43.0917 ± 0.7853
ΔE_ele_	−27.1697 ± 1.2394	−191.0526 ± 4.4944	−181.5281 ± 2.3072	−228.7382 ± 4.1515
ΔG_solv_	83.6946 ± 1.6182	209.1145 ± 3.8104	206.7081 ± 2.0331	−283.3881 ± 3.8155
ΔG_gas_	−78.3737 ± 1.4539	−219.7946 ± 4.6783	−212.3586 ± 2.2800	−271.8299 ± 4.2081
ΔG_total_	5.3209 ± 0.6830	−10.6800 ± 1.3828	−5.6505 ± 0.8874	11.5582 ± 1.6743

## 4 Discussion

The targeted computational workflow established in this study successfully bridges the gap between sequence prediction and molecular mechanism in flavor peptide discovery. By integrating LightGBM-based screening with electronic tongue evaluations and 200 ns molecular dynamics simulations, we identified highly active sunflower-derived umami peptides, notably ELLADMK and LEELMDK. These peptides exhibit intense umami profiles driven by robust electrostatic interactions and van der Waals anchoring within the T1R1/T1R3 Venus flytrap domain (VFD). Unlike traditional empirical screening, our thermodynamic and conformational analyses—particularly the induced-fit transitions observed in the free energy landscapes—provide a precise structural template for how distinct peptide sequences trigger the VFD expansion necessary for sensory signal transduction.

Despite the successful identification of potential umami peptides, this study has certain limitations regarding the in silico enzymatic hydrolysis strategy. Currently, only trypsin was utilized for the virtual digestion due to its highly specific and well-defined cleavage sites, which facilitated the initial efficient screening of target peptides. However, this single-enzyme approach differs significantly from the compound enzymatic hydrolysis widely employed in the actual food industry. Industrial food processing typically utilizes complex enzyme mixtures (such as alcalase, flavourzyme, or papain) to maximize protein degradation, enhance solubility, and accelerate flavor release. Consequently, relying solely on trypsin limits the diversity of the generated peptide pool and may overlook potentially highly active umami peptides produced by other cleavage patterns.

Furthermore, while the combination of machine learning predictions and electronic tongue sensory evaluations provides robust initial data, the current pipeline relies strictly on computational models and macroscopic sensory responses. The absence of cell-based functional assays, such as calcium imaging in taste receptor-expressing cells, limits the comprehensive validation of the physiological signal transduction pathways downstream of receptor binding.

Future studies should incorporate multi-enzyme simulated digestion models and experimental compound enzymatic validations to better bridge the gap between computational screening and practical industrial applications. Additionally, integrating *in vitro* cellular assays will be crucial to experimentally confirm the molecular dynamics findings, ultimately facilitating the targeted design and industrial-scale production of high-value flavor peptides from sustainable plant proteins.

## 5 Conclusion

In this study, an integrated AI-driven and biophysical framework was established to discover novel sunflower-derived umami peptides. Utilizing a LightGBM model based on ESM-2 features, four candidates were identified, among which ELLADMK and LEELMDK exhibited the most potent umami and richness profiles in electronic tongue evaluations. Molecular docking and 200 ns MD simulations elucidated that these peptides bind the T1R1/T1R3 receptor via robust electrostatic and hydrogen-bonding networks. Furthermore, structural and thermodynamic analyses revealed a classic induced-fit mechanism: highly active peptides, particularly ELLADMK, specifically modulate key residue flexibility and energetically lock the receptor into a stable, low-energy active conformation. This research highlights sunflower meal as a sustainable source for flavor enhancers and provides a robust paradigm for high-throughput flavor peptide discovery.

## Data Availability

The original contributions presented in the study are included in the article/Supplementary material, further inquiries can be directed to the corresponding authors.

## Footnotes

1
https://alphafoldserver.com

2
http://huanglab.phys.hust.edu.cn/hpepdock/
