# Supplementary material for: Discovery of sunflower-derived umami peptides via machine learning and molecular dynamics simulation of their interactions with the T1R1/T1R3 receptor
Source: Front Nutr. 2026 Sep 25;13:1961387. doi: 10.3389/fnut.2026.1961387 (PMC13650002; doi:10.3389/fnut.2026.1961387)
Supplement: Supplementary file 1 [file Supplementary_file_1.DOCX]

**Supplement File**

Figure S1. Secondary structure dynamics of the T1R1/T1R3 receptor.
